# *Instance of a Disease, to Which* Sauvages *Has Given the Name of Meteorismus Ventriculi; with Remarks*

**Published:** 1791

**Authors:** Robert Graves

**Affiliations:** Physician at Sherborne, in Dorsetshire; and Extra Licentiate of the College of Physicians, London.


### VIII. *Inſtance of a Diſeaſe, to which* Sauvages *has given the Name of Meteoriſmus Ventriculi; with Remarks. By* Robert Graves, *M. D. Phyſician at Sherborne, in Dorſetſhire; and Extra Licentiate of the College of Phyſicians, London.*

ON the 2d of February 1791, Ann Hunt, who had juſt entered into her fifteenth year, was affected with an uncommon, large, hard, uniform, prominent tumour or ſwelling in the epigaſtric region, extending from the ſternum to ſome diſtance below the umbilicus. It was of a circular form, accompanied with very little or no pain, excepting upon its being preſſed; and even in that caſe, the pain excited was but inconſiderable. She perceived ſome ſlight difficulty of breathing, upon uſing any bodily exertion, particularly that of walking; ſhe had much thirſt, yet her appetite for food remained tolerably good, no way depraved, and her belly regular, with but little apparent loſs of fleſh. No obſervable change in her countenance could be diſcovered, except that it had become ſomewhat of a paler colour than uſual.

The appearance of this extraordinary ſwelling was firſt perceived in the region of the ſtomach,

ſome

ſome time in the month of June 1790, of a ſize not larger than that of a hen's egg. From this time it continued gradually to increaſe, till about the commencement of the enſuing Auguſt, when the enlargement which it had received in the courſe of this interval was ſuch, that the whole ſpace between the ſternum and umbilicus became completely occupied by it. Afterwards it remained with little variation to the period of its removal. Shortly after the appearance of this ſwelling, ſome medicines were given her with a view to its diſcuſſion; but from their ſeeming inefficacy, joined to her parents' ſlender ability to procure them, on account of their expence, they were ſoon diſcontinued.

As I was ſtrongly perſuaded, from the age of the patient, and other circumſtances, accompanying the caſe, that it was to be conſidered as falling under the denomination of *meteoriſmus*, as deſcribed by Sauvages, I reſolved to ſupply her with ſuch medicines as the nature of the caſe ſeemed to me to indicate, in order to learn with greater certainty from the event, whether I was right in the judgement I had thus formed of it. Accordingly on the 2d of February, when ſhe came to me, I gave her ſome powders, conſiſting of about eighteen grains of prepared ſteel, and and directed one of them to be taken twice a day. She had likewise a purgative medicine, which was ordered to be taken early the next morning, composed of a scruple of rhubarb, and of about three grains of calomel. This powder operated briskly and well; her stools were of a blackish colour, and slightly offensive.

From this operation the size of the tumour seemed no way diminished or affected. But after a continuance of the chalybeate powders only for the short space of three or four days, her swelling was found lessened to a considerable degree; and in a day or two afterwards was totally removed, so that the parts, which before had been so exceedingly hard and protuberant, became enabled to resume their natural form, situation, and softness. It is now upwards of two months since she was happily relieved; nor has she experienced, as yet, the smallest alarm from any sign or appearance which the complaint has at all shewn of returning again. It may not be improper here to observe that, though the quantity of eighteen grains of chalybs makes a pretty large dose to begin with, and would in most tender habits be productive of some degree of sickness, it had no such stimulant effect in the case before us. The only sensible operation attendant on its exhibition was, its occafioning violent and frequent eructations of wind; and it is fomewhat remarkable, that this effect took place very foon after the firft dofe of it had been fwallowed, and before the period at which the fecond was taken.

That the cafe above mentioned affords a perfect, though uncommon example of the *meteorifmus ventriculi*, as defcribed by Sauvages, the fymptoms accompanying it, together with the means employed in its cure, bear ample and unequivocal evidence. Although this is a difeafe, which has been faid by fome authors not to be of infrequent appearance in chlorotic females, and others labouring under a fuppreffion of the menfes, yet it is prefumed that the prefent inftance, on account of the peculiarities difplayed by it, cannot be deemed altogether unworthy of regard. The flow and gradual manner in which the fwelling acquired its increafe of bulk; the enormous and extenfive fize it attained; its long continuance afterwards without any very obfervable change; and laftly its yielding fo fpeedily to the remedies made ufe of, are all of them circumftances that may render it perhaps highly acceptable to the curious and intelligent reader.

Of

Of the ſeveral particulars here mentioned the two firſt are certainly of very rare occurrence, ſeldom to be obſerved in caſes of a like nature. Whether in the ſtomach or inteſtines, when any ſwelling ariſes ſolely in conſequence of a collection of air, its riſe and progreſs are for the moſt part ſudden, and as it were inſtantaneous; and there are but few examples of its appearing firſt of a diminutive ſize, and then receiving a ſlow and gradual enlargement for any long continued portion of time. It is from a knowledge of this and other well-aſcertained facts, that we are enabled to diſtinguiſh, with ſufficient accuracy, between dropſical ſwellings and others, of the abdomen, that have nothing but an accumulation of confined air for the immediate cauſe of their diſtention. As to the enormous ſize the ſwelling exhibited, I believe it may be ſafely aſſerted, that ſuch another inſtance has ſcarce ever fallen within the eye and obſervation of the oldeſt and moſt experienced medical practitioners. In general the ſwelling in theſe caſes amounts to little more than to fill up the hollow or depreſſion between the ſternum and the umbilicus, obſervable in thoſe who are altogether exempt from any diſeaſe of thofe parts; "ita ut nulla fit cavitas ab fterno ad umbilicum qualis in fanis eft *."

The late Dr. Cullen, who has fo defervedly acquired the thanks and praifes of all thofe that are engaged in medical purfuits, for the many important fervices which have been rendered by him in almoft every branch of our fcience, comprehends this and the three other remaining fpecies of the genus, meteorifmus, of Sauvages, under the general title of Tympanites. Though there are undoubtedly obvious advantages refulting from his careful and judicious reduction of the difeafes to which mankind is liable, to a lefs number than what had been before, by rendering the ftudy and knowledge of them more eafy and expeditious; yet it is doubtful, whether in confequence thereof, fome ambiguity alfo be not incurred often in practice. No one from a perufal of the account which Dr. Cullen has given us of tympanic affections, would have fuppofed without the aid of fome additional information, that the above cafe ought to have been referred to this head of difeafes. It is much more probable that its caufe, under fuch circumftances, would have been attributed to fome morbid obftruction and enlarge-

* Sauvages Nofol. method.

ment of the liver, or of ſome other adjoining viſcus contained within the cavity of the abdomen.

From the event of the foregoing caſe we have likewiſe an excellent example of the ſuperior power of ſteel as a tonic medicine; ſince the generation of the diſtending air cannot but be ultimately reſolved into a laxity of the fibres of the ſtomach. In caſes even where the complaint is confined ſolely to the inteſtines, its uſe ſeems from hence to be particularly pointed out. The flatulent nature of theſe caſes has alſo led to the employment of medicines of the carminative claſs; but though the milder kinds may be adminiſtered without any ſuſpicion of danger, their poſſeſſing any real efficacy is extremely doubtful, and particularly where the inteſtinal canal becomes the ſeat of the diſeaſe.

